# Signaling from the Sympathetic Nervous System Regulates Hematopoietic Stem Cell Emergence during Embryogenesis

**DOI:** 10.1016/j.stem.2012.07.002

**Published:** 2012-10-05

**Authors:** Simon R. Fitch, Gillian M. Kimber, Nicola K. Wilson, Aimée Parker, Bahar Mirshekar-Syahkal, Berthold Göttgens, Alexander Medvinsky, Elaine Dzierzak, Katrin Ottersbach

**Affiliations:** 1 Department of Haematology, Cambridge Institute for Medical Research, Wellcome Trust-Medical Research Council Stem Cell Institute, University of Cambridge, Cambridge CB2 0XY, UK; 2 Erasmus Stem Cell Institute, Department of Cell Biology, Erasmus Medical Center, 3000 CA Rotterdam, The Netherlands; 3 MRC Centre for Regenerative Medicine, Institute for Stem Cell Research, University of Edinburgh, Edinburgh EH9 3JQ, UK

## Abstract

The first adult-repopulating hematopoietic stem cells (HSCs) emerge in the aorta-gonads-mesonephros (AGM) region of the embryo. We have recently identified the transcription factor Gata3 as being upregulated in this tissue specifically at the time of HSC emergence. We now demonstrate that the production of functional and phenotypic HSCs in the AGM is impaired in the absence of Gata3. Furthermore, we show that this effect on HSC generation is secondary to the role of Gata3 in the production of catecholamines, the mediators of the sympathetic nervous system (SNS), thus making these molecules key components of the AGM HSC niche. These findings demonstrate that the recently described functional interplay between the hematopoietic system and the SNS extends to the earliest stages of their codevelopment and highlight the fact that HSC development needs to be viewed in the context of the development of other organs.

## Graphical abstract

**Graphical Abstract:**

**Figure:**
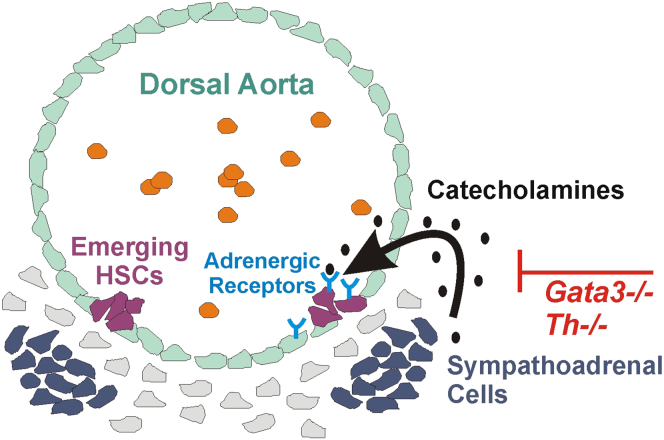

**Highlights:**

► Gata3-deficient AGMs have reduced hematopoietic stem cell (HSC) numbers ► Gata3-dependent catecholamine production regulates HSC emergence ► Catecholamines regulate HSC emergence independent of blood flow ► Nascent HSCs express catecholamine receptors

## Teaser

Catecholamines produced by the sympathetic nervous system regulate hematopoietic stem cell emergence during embryonic development.

## Introduction

The emergence of adult-repopulating hematopoietic stem cells (HSCs) initiates in the mouse embryo at embryonic day (E) 10.5 in an intraembryonic region that comprises the developing circulatory, reproductive, and excretory systems and has therefore been termed the aorta-gonads-mesonephros (AGM) region (Medvinsky and Dzierzak, 1996; Medvinsky et al., 1993; Müller et al., 1994). This tissue is of a highly dynamic and transient nature and only contains HSCs between E10.5 and E12.5, at which point the fetal liver has established itself as the main hematopoietic organ for the remainder of fetal development (Kumaravelu et al., 2002). The origin of HSCs within the AGM and the process of their generation have been the matter of intense debate over the past decade; however, recent findings have started to shed some light on these issues (for a recent review, see Ottersbach et al., 2010). Originally, two seemingly conflicting theories were put forward of either an endothelial (Jaffredo et al., 1998, 2000) or a subaortic mesenchymal (Bertrand et al., 2005) precursor cell for HSCs. Recent lineage tracing experiments have reported the existence of hemogenic endothelial cells in the mouse AGM that contribute to the production of definitive blood both in the embryo and in the adult (Chen et al., 2009; Zovein et al., 2008). However, there is also evidence for the existence of an early subaortic mesenchymal population that expresses smooth muscle cell markers and that contributes to blood formation via an endothelial intermediate, thus providing a possible link between the two original hypotheses (Rybtsov et al., 2011; Zovein et al., 2008). There is also recent evidence that the yolk sac may contribute to HSC precursors (Samokhvalov et al., 2007; Tanaka et al., 2012).

Progress has also been made in defining the components that make up the hematopoietic regulatory microenvironment of the AGM. Cells with characteristics of mesenchymal stromal cells, which are known to be crucial elements in the adult HSC niche, have been identified in the AGM (Mendes et al., 2005), and AGM-derived stromal cell lines have proven to be a powerful tool for the identification of environmental HSC regulators (Durand et al., 2007; Ohneda et al., 2000; Oostendorp et al., 2005; Renström et al., 2009). Furthermore, it was demonstrated that tissues ventral to the AGM have an enhancing effect on HSC emergence, while tissues on the dorsal side decrease HSC production (Peeters et al., 2009; Taoudi and Medvinsky, 2007), pointing to the important concept that HSC generation in the AGM occurs in the wider context of the tissues that develop around it.

We have recently carried out microarray expression analyses of the AGM region with the aim of identifying HSC regulators (Mascarenhas et al., 2009). In addition to known hematopoiesis-associated genes, upregulation of genes known to be involved in the development of tissues surrounding the aorta was also noted, again suggesting that HSC emergence is functionally linked to other developmental processes that proceed in the vicinity of the dorsal aorta.

Among the upregulated genes was the transcription factor *Gata3*, which is involved in the development of a number of cell and tissue types, including skin, mammary gland, kidney, sympathetic nervous system (SNS), and adipocytes (Asselin-Labat et al., 2007; Grote et al., 2006; Kaufman et al., 2003; Kouros-Mehr et al., 2006; Kurek et al., 2007; Pandolfi et al., 1995; Tong et al., 2000). Within the hematopoietic system, Gata3 has been linked to the development of T cells (Ting et al., 1996) and thymic natural killer cells (Vosshenrich et al., 2006). Moreover, Gata3 expression was reported in embryonic and adult HSCs (Benveniste et al., 2010; Bertrand et al., 2005; Kent et al., 2009) and the knockout embryos were shown to have defective fetal liver hematopoiesis (Pandolfi et al., 1995). More recently, two groups have confirmed Gata3 expression in adult HSCs and have examined a possible role for Gata3 in adult HSC function. While Buza-Vidas et al. have found no effect of a panhematopoietic Gata3 deletion on the ability of HSCs to long-term repopulate and self-renew (Buza-Vidas et al., 2011), Ku et al., using a complete *Gata3* null line and an adult hematopoiesis- and bone-marrow-niche-specific *Gata3* knockout, reported that Gata3 is required for adult HSC maintenance via regulation of cell cycle entry (Ku et al., 2012).

Considering the reported role of Gata3 in the hematopoietic system and its importance in the development of various other tissues, we hypothesized that it may also be involved in the generation of the first HSCs, especially since we previously found Gata3 to be upregulated in the E11 AGM. We therefore analyzed Gata3-deficient embryos and detected a marked reduction in functional and phenotypic HSCs in the AGM. Surprisingly, this was largely independent of a cell-intrinsic role of Gata3, but was secondary to its function in the developing SNS, thus providing an example of a molecular defect in an adjacent tissue having a profound impact on HSC production in the dorsal aorta.

## Results

### *Gata3* Deletion Causes a Reduction in Hematopoietic Stem and Progenitor Cells in E11.5 and E10.5 AGMs

To determine if Gata3 plays a role in the emergence of HSCs, we analyzed the repopulation activity of AGMs from *Gata3* knockout embryos in transplantation assays. When E11–11.5 AGMs were dissected from *Gata3*^*+/+*^, *Gata3*^*+/−*^, and *Gata3*^*−/−*^ embryos and directly transplanted into adult recipients, a marked reduction in the repopulation ability of *Gata3*^*+/−*^ and *Gata3*^*−/−*^ AGMs was observed (Table 1 and Figure S1 available online). While almost 60% of mice transplanted with wild-type AGM cells showed more than 10% donor contribution to their peripheral blood, less than 20% of mice were repopulated by *Gata3*^*+/−*^ and *Gata3*^*−/−*^ AGM-derived HSCs. In fact, only one mouse out of seven injected with *Gata3*^*−/−*^ AGM cells had detectable levels of donor contribution, and these did not exceed 10%, even after more than 4 months. Placing E11 AGMs into whole-organ explant culture for 3 days prior to transplantation allowed HSC activity in heterozygous AGMs to recover to that of wild-type levels, while the defect remained in *Gata3* null AGMs (Table 1 and Figure S1). These results suggest that reduced levels of Gata3 impair the emergence and/or expansion of HSCs in the AGM.

HSC production in the AGM initiates at E10.5 (Müller et al., 1994). However, numbers of HSCs are limited at that time point, making an accurate comparison of HSCs in E10.5 wild-type or Gata3-deficient AGMs by direct transplantations impossible. We therefore analyzed the number of immature late-developing colony-forming units-spleen (CFU-S_11_) progenitors (Figure S1). While all of the wild-type and heterozygous E10.5 AGMs contained CFU-S_11_ cells, only three out of eight injected *Gata3*^*−/−*^ AGMs produced spleen colonies in the recipients. Moreover, in those three AGMs, the frequency of CFU-S_11_ cells was less than half of that observed in *Gata3*^*+/+*^ and *Gata3*^*+/−*^ E10.5 AGMs. This implies that Gata3 plays a role in the production of hematopoietic progenitors and the first HSCs that emerge in the midgestation embryo.

### Intra-Aortic Clusters and Phenotypic HSCs Are Reduced in E11 *Gata3*^*−/−*^ Aortae

**Figure 1:**
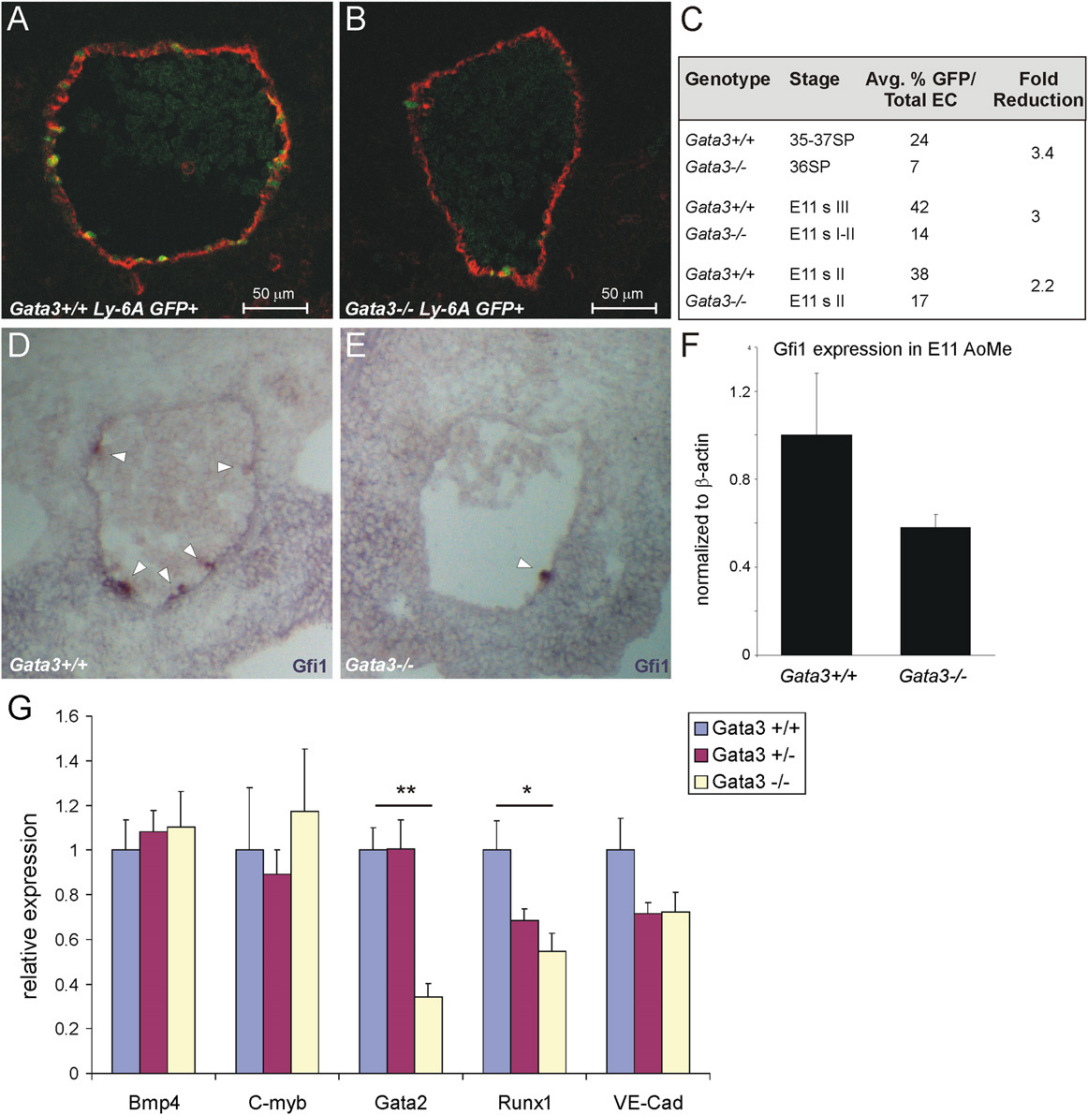
Phenotypic HSCs Are Reduced in Gata3-Deficient Embryos Sections were prepared from E10.5–E11.5 *Gata3*^*+/+*^*Ly-6A GFP+* and *Gata3*^*−/−*^*Ly-6A GFP+* embryos that had been staged according to their somite pairs (E10.5) or according to the pigmentation around their eyes (E11). Sections were costained for CD34 and the number of GFP+ cells within the total endothelial cells were counted on confocal images of every tenth section. Representative sections of *Gata3*^*+/+*^*Ly-6A GFP+* and *Gata3*^*−/−*^*Ly-6A GFP+* embryos are shown in (A) and (B), respectively. GFP, green; CD34, red/Cy5; ventral, down. A summary of the results from three independent experiments is shown in (C). SP, somite pairs; s, stage. Sections of E11 *Gata3*^*+/+*^ (D) and *Gata3*^*−/−*^ (E) embryos were stained with a riboprobe for *Gfi1*. Ventral, down, 10×/0.25 objective. Levels of *Gfi1* were determined in subdissected E11 *Gata3*^*+/+*^ and *Gata3*^*−/−*^ aorta-mesenchyme by quantitative RT-PCR (F). Error bars represent SD. (G) Quantitative RT-PCR was performed on cDNA prepared from ventral halves of aorta-mesenchymes from E11 *Gata3*^*+/+*^ (n = 3), *Gata3*^*+/−*^ (n = 7), and *Gata*^*−/−*^ (n = 3) embryos for the named genes and was normalized to *Actb* and *Tbp*. ^∗^p < 0.05, ^∗∗^p < 0.01; error bars represent the SEM of the normalized relative expressions.

To analyze HSCs phenotypically, we crossed the *Gata3* knockout line with the *Ly-6A GFP* transgenic mouse line, which expresses the green fluorescent protein (GFP) under the regulatory elements of the HSC marker Sca1 and which was previously shown to mark all HSCs in the AGM (de Bruijn et al., 2002). Sections were prepared from *Gata3*^*+/+*^ *GFP+* and *Gata3*^*−/−*^ *GFP+* embryos along the whole length of the AGM region, and the number of GFP+ cells within the endothelial layer of the dorsal aorta was counted on every tenth section. This method was previously used to highlight a reduction in phenotypic HSCs in *Gata2*^*+/−*^ embryos (Ling et al., 2004). In three independent experiments, we found a 2- to 3-fold reduction in the percentage of Ly-6A GFP+ cells within the endothelium of E10.5 and E11.5 *Gata3*^*−/−*^ aortae (Figures 1A–1C). The total number of endothelial cells was not affected by the Gata3 deficiency (data not shown). Representative sections are shown in Figures 1A and 1B. These results suggest that Gata3 deletion results in fewer phenotypic HSCs and possibly also a reduction in hemogenic endothelial cells, which are also thought to express the *Ly-6A GFP* transgene (de Bruijn et al., 2002).

We also examined the presence and morphology of intra-aortic clusters, which are believed to be signs of HSC emergence in the dorsal aorta. Sections from E11 *Gata3*^*+/+*^ and *Gata3*^*−/−*^ embryos were stained for the expression of the transcription factor growth factor independence 1 (*Gfi1*), which is known to be expressed in adult HSCs and to be required for their maintenance (Hock et al., 2004; Khandanpour et al., 2010). Figure 1D shows that *Gfi1* is strongly and specifically expressed in the intra-aortic clusters of E11 wild-type embryos, as reported previously (Wilson et al., 2010). This specific expression pattern of *Gfi1* was then used to demonstrate that the number and the size of intra-aortic clusters is reduced in *Gata3*^*−/−*^ embryos (Figures 1D and 1E), which was also shown quantitatively (Figure 1F). We looked for changes in expression of other known HSC markers by quantitative RT-PCR analysis of ventral halves of subdissected aorta-mesenchyme tissue. While *Bmp4* and *C-myb* levels were not affected by the absence of Gata3, there was a significant reduction in *Runx1* and *Gata2* expression (Figure 1G). These results validate that phenotypic HSCs, as detected by the expression of Ly-6A GFP, are reduced in AGMs deficient for Gata3 and that their production may be disrupted, as evidenced by the diminished number of hemogenic endothelial cells and intra-aortic clusters.

### Gata3 Is Expressed in Various Cell Types in the E10 and E11.5 AGM

**Figure 2:**
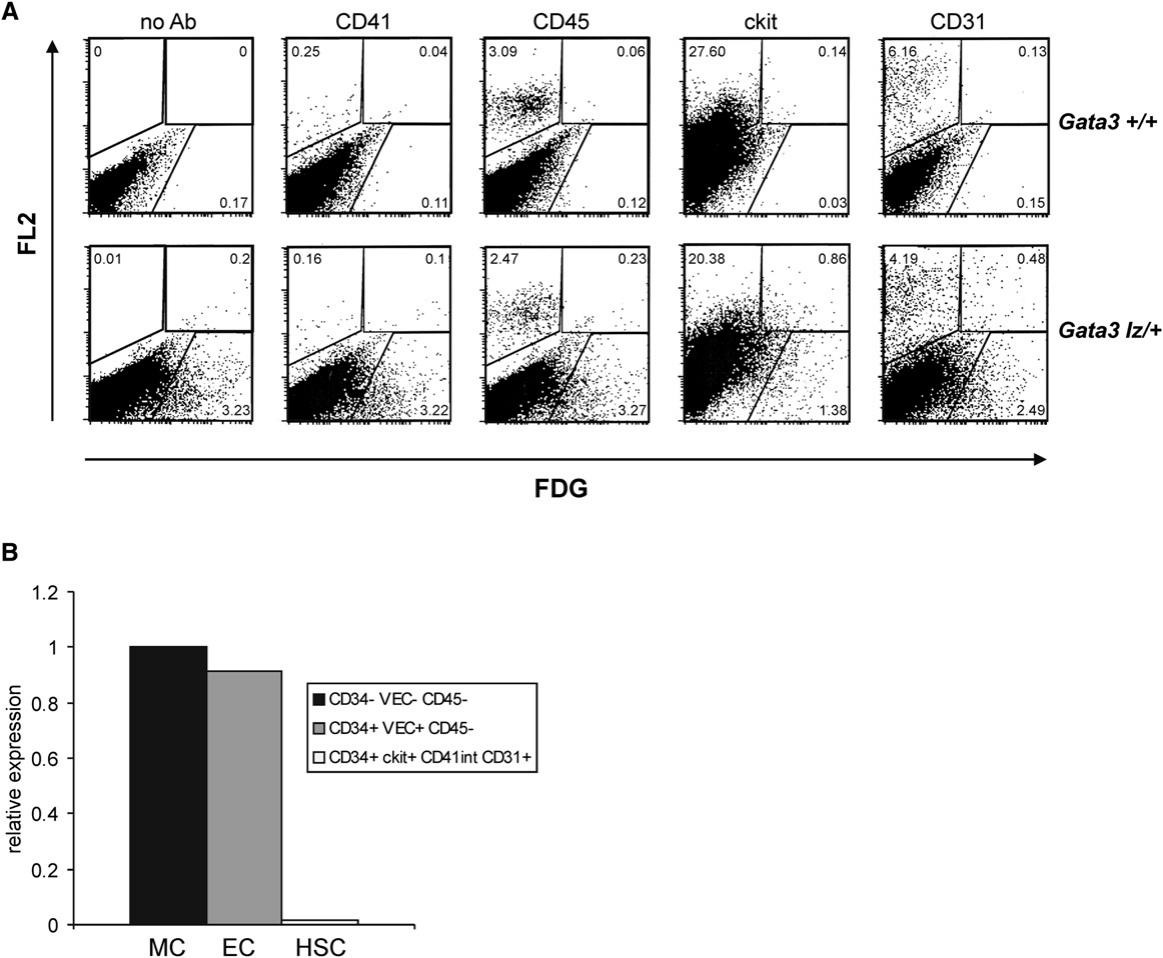
Gata3 Is Not Expressed in Hematopoietic Cells in the E11.5 AGM Region (A) *Gata3*^*+/+*^ (top panels) and *Gata3*^*lz/+*^ (bottom panels) E11.5 AGM cells were loaded with the fluorescent β-galactosidase substrate FDG and analyzed for coexpression of CD41, CD45, ckit, and CD31 (FL2). (B) Mesenchymal (MC), endothelial (EC), and hematopoietic stem cell (HSC) populations were sorted from E11.5 wild-type aorta-mesenchymes using the indicated marker combinations and the levels of *Gata3* transcripts (normalized to *Actb* and *Tbp*) determined by quantitative RT-PCR.

To understand the mechanism by which Gata3 regulates HSC production in the AGM, its expression in specific cell types was determined using a *Gata3-LacZ* knockin mouse line (van Doorninck et al., 1999). Flow cytometry analysis of FDG-loaded cells from E11.5 *Gata3*^*+/lz*^ AGMs revealed Gata3 expression in some ckit+ and some CD31+ cells (Figure 2A). However, when compared with the background control (no antibody), there does not seem to be any overlap between Gata3-expressing cells and CD41+ or CD45+ cells, suggesting that Gata3 is not expressed in hematopoietic cells in the AGM. Furthermore, isolating cell populations enriched for HSCs, endothelial cells, and mesenchymal cells from E11.5 wild-type subdissected aorta-mesenchymes by flow sorting using a combination of well-established markers (North et al., 2002; Robin et al., 2011; Sánchez et al., 1996) and analyzing these by quantitative RT-PCR showed that there is little to no *Gata3* expression in phenotypic HSCs (Figure 2B).

To further investigate if Gata3 is expressed in HSCs, Gata3-LacZ+ and LacZ− cells were tested for repopulation activity in transplantation assays. In the first set of experiments, LacZ+ and LacZ− cells were sorted from freshly dissected E11.5 *Gata3*^*+/lz*^ AGMs (the urogenital ridges were removed prior to sorting to eliminate LacZ+ mesonephric duct cells) and directly injected into recipients. However, none of the 14 mice injected with LacZ+ cells showed any signs of repopulation, while only 1 out of 13 recipients was robustly repopulated with Gata3-LacZ− cells (Table 2). This low level of HSC activity in both cell populations is most likely due to the heterozygous background of the *Gata3*^*+/lz*^ AGMs because we observed reduced repopulation activity in direct transplantation assays with total AGM cells from *Gata3*^*+/−*^ embryos (Table 1). We therefore cultured E11.5 *Gata3*^*+/lz*^ AGMs as whole-organ explants for 3 days in the presence of Interleukin 3, which has previously been demonstrated to expand AGM HSCs (Robin et al., 2006). At the end of the culture period, LacZ+ and LacZ− cells were sorted and analyzed in transplantation assays. The results in Table 2 clearly show that all of the long-term repopulating activity resides within the Gata3-LacZ− fraction, with all of the recipients that received LacZ− cells displaying high levels of donor chimerism, while none of the recipients of LacZ+ cells showed any signs of repopulation.

**Figure 3:**
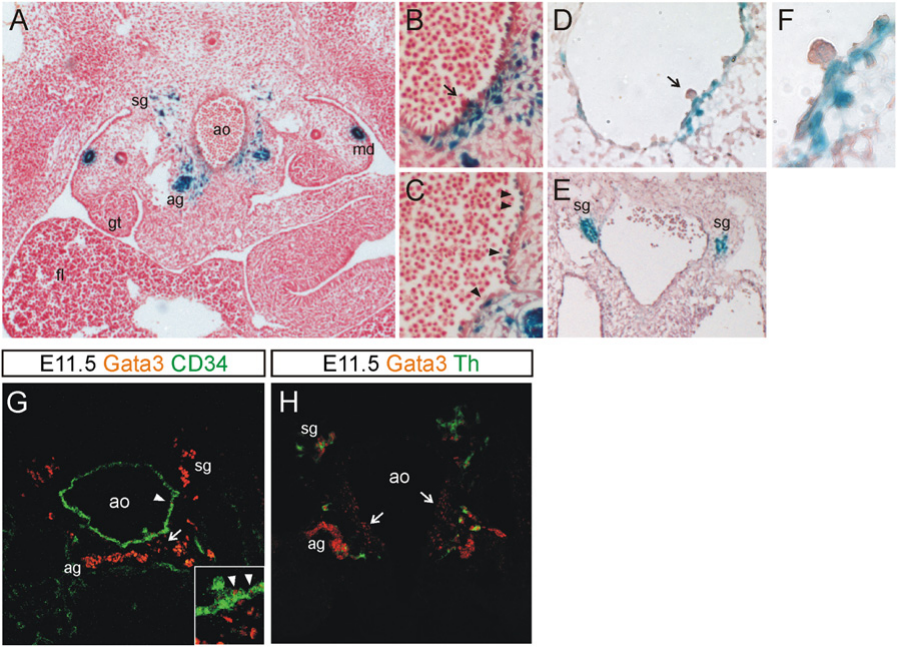
Gata3 Is Expressed in a Number of Different Cell Types in the AGM E11.5 (A–C) and E10 (D–F) *Gata3*^*lz/+*^ embryos were stained with X-gal (blue) and cryosections were counterstained with Neutral Red. Ventral, down. Objectives were 10×/0.25 (A), 20×/0.45 (B, C, and E), 40×/0.65 (D), or 100×/1.4 (F). Arrows in (B) and (D) point to intra-aortic clusters; arrowheads in  (C) highlight Gata3-expressing endothelial cells. (F) A close-up of the cluster in (D). (G and H) E11.5 wild-type sections were costained for Gata3 (red/Cy5) and CD34 (green/FITC) (G) or Th (green/Alexa 488) (H) and confocal images were obtained. Arrows in (G) and (H) point to Gata3 staining in subaortic mesenchymal cells, and arrowheads in (G) point to Gata3+ endothelial cells. ag, adrenal anlage; ao, dorsal aorta; fl, fetal liver; gt, gonadal tissue; md, mesonephric duct; sg, sympathetic ganglia; Th, tyrosine hydroxylase.

To analyze the spatial distribution of Gata3-expressing cells within the AGM, we prepared sections from X-gal-stained *Gata3*^*+/lz*^ embryos. As previously described, Gata3 expression was detected in the mesonephric ducts (Figure 3A), which is in line with its reported function in the developing kidney (Grote et al., 2006). Gata3 expression was also observed in sympathoadrenal progenitors, which form the developing adrenal gland and sympathetic ganglia of the SNS (Huber, 2006), the development of which is known to be Gata3 dependent (Lim et al., 2000; Moriguchi et al., 2006; Tsarovina et al., 2004). Upon closer inspection of the aortic region, we also saw additional Gata3 staining in subendothelial cells underneath the lateral and ventral aspect of the dorsal aorta (Figures 3B and 3C). This pattern overlaps with the subaortic patches that have previously been described (Bertrand et al., 2005; Manaia et al., 2000) and may also coincide with the compartment where other known HSC regulators, such as Bmp4 and Runx1, are expressed (Durand et al., 2007; North et al., 1999). This staining was particularly prominent underneath intra-aortic clusters (arrow in Figure 3B). Importantly, we did not detect any Gata3 expression in the clusters themselves or in any other hematopoietic cells, as suggested from the flow cytometry results in Figure 2. Some isolated endothelial cells were found to be Gata3-LacZ+ (arrowheads in Figure 3C), which again confirms our flow cytometry analysis results that showed some overlap between Gata3-LacZ and CD31 expression (Figure 2A). The observed Gata3 expression pattern was verified by immunohistochemistry, which confirmed Gata3 expression in the SNS (as evidenced by the overlap with the sympathoadrenal marker tyrosine hydroxylase, Th, Figure 3H) with additional staining observed in the subaortic mesenchyme (arrows in Figure 3H), especially underneath intra-aortic clusters (arrow in Figure 3G), and in endothelial cells (arrowheads in Figure 3G).

We also extended our analysis to E10 embryos, which again showed subendothelial Gata3 expression, albeit in fewer cells, in the vicinity of intra-aortic clusters (Figures 3D and 3F). At this stage, sympathoadrenal cells are restricted to two lateral patches on either side of the dorsal aorta (Figure 3E). Taken together, these results suggest that Gata3 may regulate AGM HSCs in a non-cell-autonomous fashion.

### Gata3 Regulates HSC Numbers via the Production of Catecholamines

**Figure 4:**
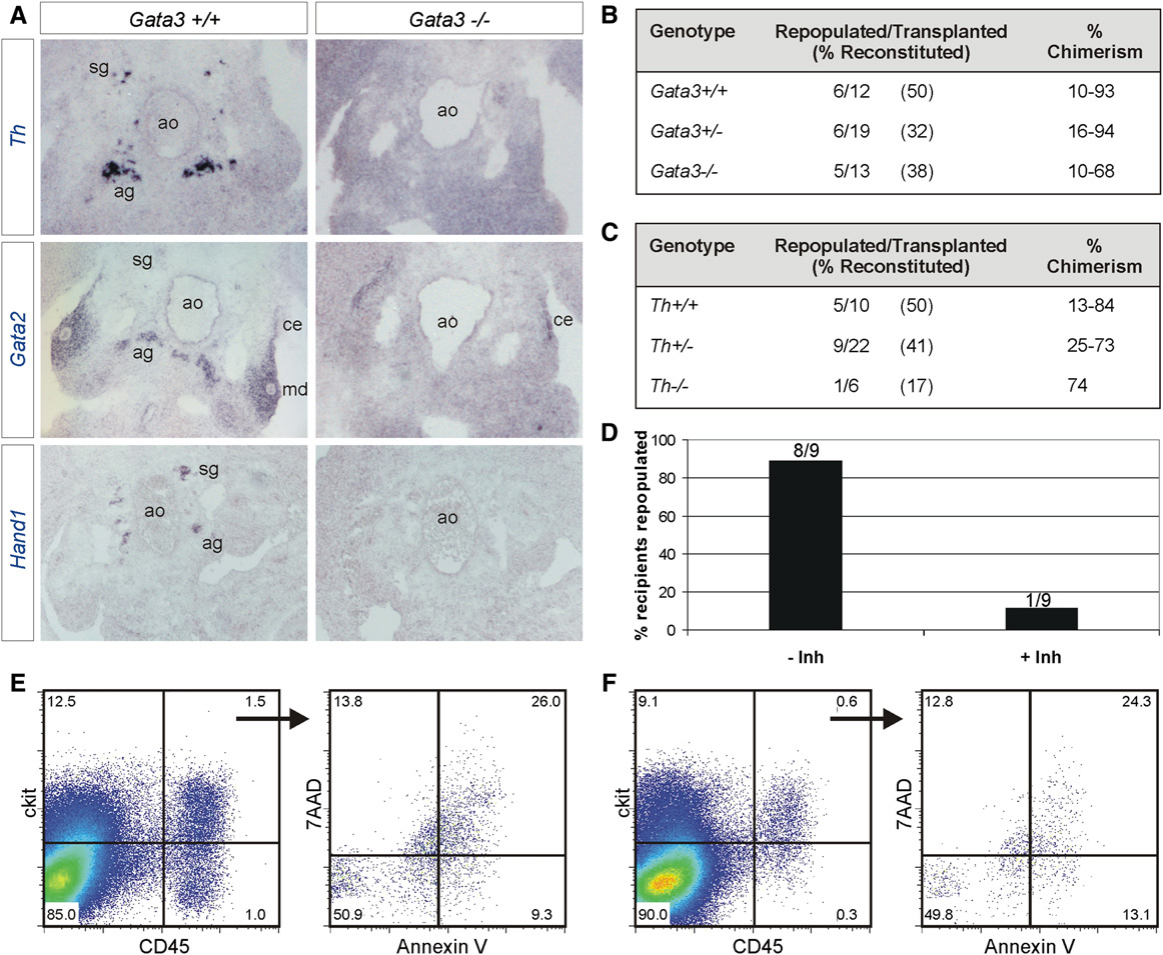
Gata3 Regulation of AGM HSCs Is Secondary to Its Role in the Sympathetic Nervous System (A) In situ hybridization with riboprobes for *Th*, *Gata2*, and *Hand1* on *Gata3*^*+/+*^ and *Gata3*^*−/−*^ E11.5 embryo sections. (B) Summary of repopulation analysis of recipients injected with uncultured E11/11.5 AGM cells (1 ee) from *Gata3*^*+/+*^, *Gata3*^*+/−*^, and *Gata3*^*−/−*^ embryos that had received catecholamine derivatives in vivo through the drinking water from E8.5. See also Figure S2. (C) Summary of repopulation analysis of recipients injected with uncultured E11/11.5 AGM cells (1 ee) from *Th*^*+/+*^, *Th*^*+/−*^, and *Th*^*−/−*^ embryos. See also Figure S2. (D) Percentage of recipients repopulated with wild-type E11.5 AGMs that had been cultured in the presence or absence of a Th inhibitor. The number of repopulated/total recipients is indicated above each bar. (E and F) Flow cytometry analysis of ckit and CD45 expression on cells from E11.5 wild-type AGMs cultured in the absence (E) or presence (F) of a Th inhibitor. The percentage of apoptotic cells within the ckit+CD45+ population is shown.

As mentioned above, Gata3 is essential for the formation of the SNS (Lim et al., 2000; Moriguchi et al., 2006; Tsarovina et al., 2004, 2010). We confirmed this by comparing the expression pattern of three genes that are active in cells of the developing SNS, in sections of wild-type and *Gata3*^*−/−*^ embryos. These genes included *Th*, an enzyme required for the biosynthesis of catecholamines, the mediators of the SNS; the transcription factor *Gata2*, which is known to lie downstream of Gata3 in the development of the SNS (Tsarovina et al., 2004); and the transcription factor *Hand1* (Cserjesi et al., 1995; Morikawa and Cserjesi, 2004). *Th* and *Hand1* expression are restricted to sympathoadrenal cells in wild-type AGMs and are completely absent in *Gata3*^*−/−*^ AGMs (Figure 4A). In addition to its expression in sympathoadrenal cells, *Gata2* is also expressed in the urogenital ridges in the tissue surrounding the mesonephric ducts, in the coelomic epithelium, and in the endothelium of the dorsal aorta (Figures 4A and S2A). Its expression in the SNS is completely abrogated in *Gata3*^*−/−*^ embryos. Interestingly, *Gata2* is also reduced around the mesonephric ducts, suggesting that it may also act downstream of Gata3 in kidney development. There were also fewer Gata2-expressing cells in the endothelium of *Gata3*^*−/−*^ embryos as well as fewer Gata2+ intra-aortic clusters, thus providing further evidence for a disruption in HSC production in the AGM (Figure S2A).

The dual role of Gata3 in the developing hematopoietic system and SNS is intriguing in the light of recent reports that have demonstrated a role for the SNS in adult HSC mobilization, proliferation, and repopulation (Katayama et al., 2006; Méndez-Ferrer et al., 2008; Spiegel et al., 2007). It led us to hypothesize that such a functional link between these two systems might also exist during their development and that the function of Gata3 in the emergence of HSCs in the AGM may be secondary to its role in the developing SNS. *Gata3*^*−/−*^ embryos die at E11.5 due to a deficiency in the production of catecholamines from sympathoadrenal cells (Lim et al., 2000; Moriguchi et al., 2006). This early lethality can be delayed with a pharmacological rescue protocol in which α- and β-adrenergic agonists are supplied to the embryos via the drinking water of the pregnant dams (Lim et al., 2000; Pattyn et al., 2000). If, in analogy with their role in the adult hematopoietic system, catecholamines also regulate HSCs in the AGM, supplying the Gata3-deficient embryos with an external source of these mediators should also restore AGM hematopoiesis. We therefore started the rescue protocol at E8.5, obtained AGMs from these embryos at E11.5, and transplanted them directly. This led to a marked recovery of HSC activity in both heterozygous and *Gata3* null AGMs with the percentage of repopulated recipients having more than doubled (compare direct transplantation results in Table 1 with Figure 4B). The reconstitution levels obtained in the recipients repopulated with *Gata3*^*−/−*^ AGMs were also markedly improved (Figure 4B and Figure S2B). This suggests that the HSC defect observed in E11 Gata3-deficient AGMs is indeed secondary to the role Gata3 plays in the developing SNS. To provide further evidence we also determined HSC activity in Th knockout mice, in which the only deficiency is the absence of catecholamine production. Indeed, there was a similar reduction in repopulation activity in *Th*^*−/−*^ and *Gata3*^*−/−*^ AGMs (compare Figure 4C with Table 1 and Figure S1 with Figure S2C). Furthermore, we were also able to produce a dramatic reduction in HSCs by adding a Th inhibitor to AGM explant cultures (Figure 4D). Flow cytometry analysis showed that this was due to a decrease in the production of ckit+CD45+ cells because there was no increase in the number of apoptotic cells within that population (Figures 4E and 4F).

### The Effect of Catecholamines on Developing HSCs Is Independent of Blood Flow

**Figure 5:**
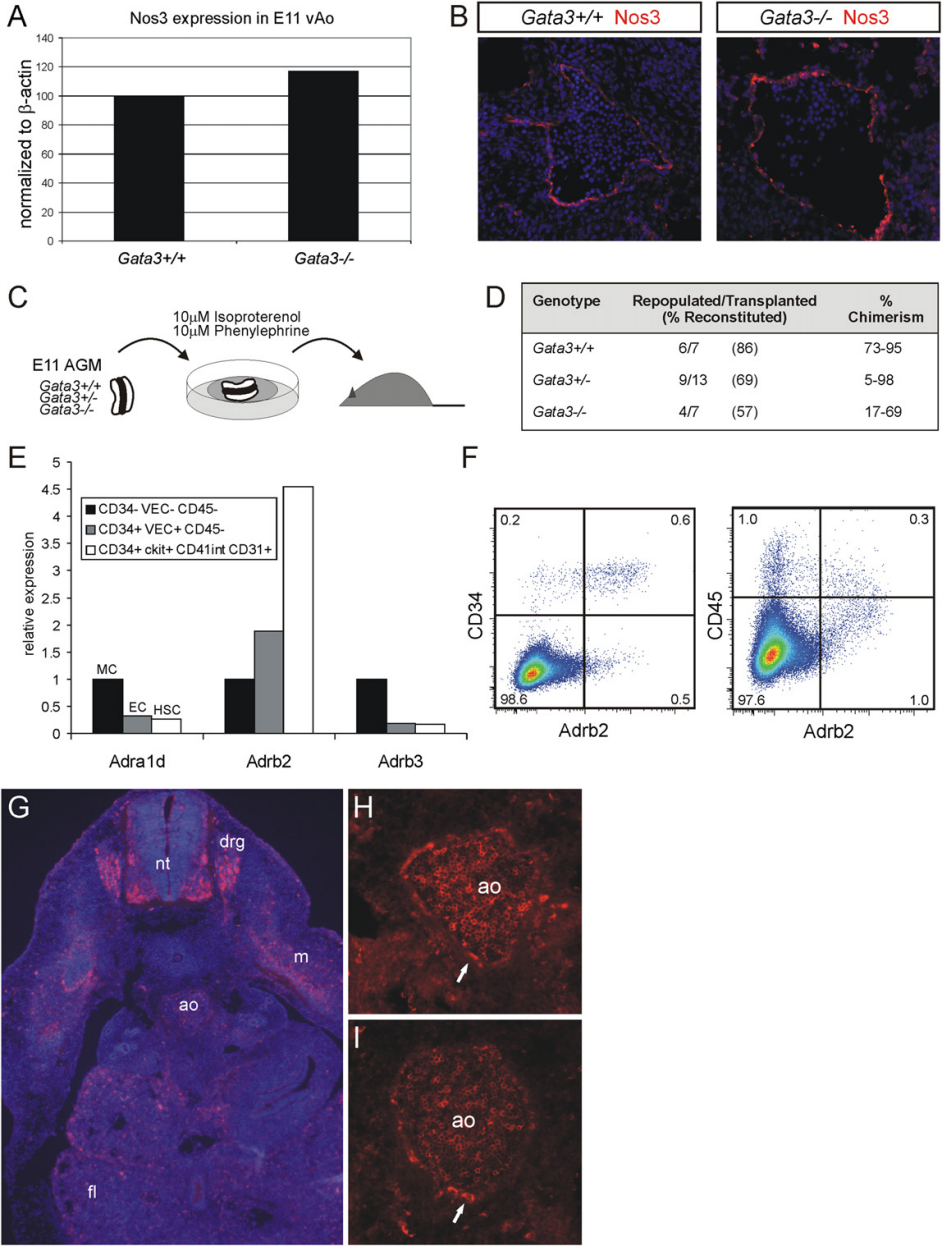
Catecholamines Can Rescue HSC Activity In Vitro in the Absence of Circulation (A) Ventral halves of dorsal aortae from E11 *Gata3*^*+/+*^ and *Gata3*^*−/−*^ embryos were analyzed for *Nos3* expression by quantitative RT-PCR. Data is representative of two independent experiments. (B) E11.5 *Gata3*^*+/+*^ and *Gata3*^*−/−*^ embryo sections were stained for Nos3 (red/Alexa 555) and counterstained with DAPI (blue). Ventral, down; 20×/0.45 objective. (C) Schematic outline of catecholamine treatment of AGMs in explant culture. (D) Summary of repopulation analysis of recipients injected with cells (1 ee) from *Gata3*^*+/+*^, *Gata3*^*+/−*^, and *Gata3*^*−/−*^ E11/11.5 AGMs that had been exposed to catecholamine derivatives in explant cultures. See also Figure S3. (E) Quantitative RT-PCR expression analysis of the α1d- (*Adra1d*), β2- (*Adrb2*), and β3- (*Adrb3*) adrenergic receptors in mesenchymal (MC), endothelial (EC), and hematopoietic stem cell (HSC) populations sorted from E11.5 aorta-mesenchymes (normalized to *Actb* and *Tbp*). (F) Flow cytometry analysis of the expression of the β2-adrenergic receptor on CD34+ or CD45+ E11.5 AGM cells. (G–I) Immunohistochemistry on E11.5 wild-type embryo sections with an antibody to the β2-adrenergic receptor (red, Cy3). Nuclear DAPI staining is shown (blue) in (G). Arrows in (H) and (I) highlight expression on endothelial cells. Ventral, down; objectives were 10×/0.25 (G) or 20×/0.45 (H and I). ao, dorsal aorta; drg, dorsal root ganglia; fl, fetal liver; m, myotome; nt, neural tube.

Based on the phenotype of embryos deficient for Th or dopamine β-hydroxylase, two enzymes involved in the synthesis of catecholamines, it was suggested that catecholamines are necessary for proper cardiovascular function and the ability to maintain a sufficient heart rate (Thomas et al., 1995; Zhou et al., 1995). Interestingly, it was recently demonstrated that a heartbeat and the sheer stress induced by a robust blood flow are needed for the generation of HSCs in the dorsal aorta via the production of nitric oxide (Adamo et al., 2009; North et al., 2009). An interpretation of our results may therefore be that the absence of catecholamines in the Gata3-deficient embryos has a negative impact on blood flow and reduces the sheer stress that hemogenic endothelial cells are exposed to in the aorta, thus leading to defective HSC production. To determine whether the absence of Gata3 leads to a reduction in sheer stress and diminished nitric oxide production, we analyzed the expression of the nitric-oxide-synthesizing enzyme Nos3 because it is known to be required for HSC production in the AGM (North et al., 2009). Figures 5A and 5B clearly show that *Nos3* levels in the floor of the dorsal aorta are not reduced in the absence of Gata3, implying that Gata3 deficiency does not cause a reduction in the blood flow through the aorta.

To further demonstrate that the effect of catecholamines on HSC production in the AGM is indeed independent of the presence of a robust blood flow, we investigated whether catecholamines retained their ability to rescue the HSC deficiency in *Gata3*^*−/−*^ embryos in an in vitro AGM explant culture system (Figure 5C). E11 AGMs were dissected from *Gata3*^*+/+*^, *Gata3*^*+/−*^, and *Gata3*^*−/−*^ embryos, cultured in the presence of catecholamine derivatives at concentrations previously shown to be effective in vitro (Méndez-Ferrer et al., 2008; Toda et al., 2007; Wei et al., 2007) and transplanted into irradiated recipients. The results in Figure 5D demonstrate that exposure of AGMs to catecholamines in vitro can indeed restore HSC levels in *Gata3* null AGMs, with the number of repopulated mice equaling the number repopulated with wild-type AGMs in the absence of catecholamines (compare Figure 5D with Table 1). The effect of catecholamines in vitro seems to be even more pronounced than when they were administered in vivo (compare Figure 5D with Figure 4B). In fact, even *Gata3*^*+/+*^ and *Gata3*^*+/−*^ AGMs exposed to catecholamines in vitro demonstrated an increased repopulation potential (compare Figure 5D with Figure 4B and Table 1). This increase in repopulation potential in in-vitro-treated AGMs was also reflected in the donor chimerism of individual (repopulated) mice (compare Figure S3 with Figure S1). This shift toward higher repopulation levels in individual mice was already to some extent observed with recipients that had been injected with AGMs treated with catecholamines in vivo (Figure S2B).

### Adrenergic Receptors Are Present on Nascent HSCs

Both the β_2_- (Adrb2) and the β_3_- (Adrb3) adrenergic receptors have been proposed to relay the effect of the SNS on adult HSCs. The β_2_-adrenergic receptor was shown to be expressed on the surface of HSCs (Spiegel et al., 2007), thus allowing the SNS to act directly on stem cells, while the β_3_-adrenergic receptor was reported to be expressed on bone marrow stromal cells (Méndez-Ferrer et al., 2008), implying that SNS-mediated HSC mobilization occurred indirectly via the niche. We analyzed the expression of these two receptors in sorted populations of mesenchymal, endothelial, and hematopoietic stem cells from the E11.5 aorta-mesenchyme region by quantitative RT-PCR (Figure 5E). Since the rescue protocol also includes an α_1_-adrenergic receptor agonist and the catecholamines adrenaline and noradrenaline can also bind to the α_1_-adrenergic receptor (Adra1d), we included this receptor in the same expression analysis. Only the β_2_-adrenergic receptor displayed high levels of expression in HSCs (Figure 5E). Flow cytometry analysis confirmed that it is expressed on a subset of CD34+ (75%) and CD45+ (23%) cells (Figure 5F). We therefore further analyzed the localization of the β_2_-adrenergic receptor by immunohistochemistry on E11.5 embryo sections and detected it on cells of the neural tube, the dorsal root ganglia, the myotome, the fetal liver, and the dorsal aorta (Figure 5G). Interestingly, within the aorta we saw expression of the receptor on numerous blood cells in the circulation, on some endothelial cells (arrow in Figure 5H), and on more rounded cells in the ventral endothelium (arrow in Figure 5I). This expression pattern suggests that catecholamines may be acting directly on HSCs or their precursors and also on more mature blood cells in the circulation.

## Discussion

We have demonstrated that a deficiency in Gata3 leads to a marked reduction in the production of HSCs in the AGM. Our data suggest that Gata3 deficiency disrupts HSC development through its role in directing expression of *Th* and hence catecholamine production in cells of the SNS, thus making the SNS an important component of the developing HSC microenvironment.

The HSC defects in Gata3-deficient embryos are dose dependent. In direct transplantations, *Gata3* haploinsufficient AGM cells show a reduction in HSC activity that is similar to what we observed with homozygous null AGM cells. Interestingly, when haploinsufficient AGMs were cultured as whole organ explants for 3 days prior to transplantation, HSC activity recovered to wild-type levels. This could be explained by the possibility that, unlike in *Gata3*^*−/−*^ embryos, functional HSCs are present in *Gata3*^*+/−*^ E11 AGMs, albeit in reduced numbers, and these HSCs have the capacity to expand during the culture step. Additionally, the explant culture medium may contain traces of catecholamines that are enough to rescue the defect in the *Gata3*^*+/−*^ AGMs, but not in the *Gata3*^*−/−*^ AGMs. To test this, we performed an ELISA for adrenaline and noradrenaline on the explant culture medium and indeed found traces of these two catecholamines (5 nM adrenaline and 1 nM noradrenaline). CFU-S numbers in the E10.5 AGM seem to be less sensitive to the dose of Gata3 because their numbers are normal in *Gata3*^*+/−*^ embryos. This may hint at a different requirement for Gata3 in HSCs compared with that in CFU-S.

Despite reports of Gata3 expression in immature *Rag1γc*^*−/−*^-repopulating cells, in primitive HSCs in human cord blood, and in adult HSCs (Benveniste et al., 2010; Bertrand et al., 2005; Kent et al., 2009; Panepucci et al., 2009, 2010), we were unable to detect robust levels of *Gata3* expression in hematopoietic cells or phenotypic HSCs sorted from the AGM. Furthermore, all of the repopulating activity in the E11.5 AGM was found in the Gata3-LacZ− cell fraction. While we cannot rule out that there is some Gata3 expression in a rare subset of AGM HSCs, in which it might perform a function similar to what has been described for adult HSCs (Ku et al., 2012), these results may also suggest that Gata3 plays a non-cell-autonomous role during the emergence of HSCs during development. This microenvironmental role of Gata3 in HSC generation would also explain why *Gata3*^*−/−*^ ESCs were able to contribute to all hematopoietic lineages, apart from T cells, in blastocyst chimeras when a wild-type microenvironment is provided (Ting et al., 1996).

To determine the component of the HSC regulatory microenvironment that is affected by the *Gata3* deletion, we concentrated on the SNS because Gata3 has a well-documented role in sympathoadrenal lineage development (Lim et al., 2000; Moriguchi et al., 2006; Tsarovina et al., 2004, 2010). Intriguingly, a rescue of the SNS defect in Gata3-deficient embryos by the external supply of catecholamines also resulted in the restoration of HSC activity in E11 *Gata3*^*−/−*^ AGMs. Furthermore, catecholamines were also able to rescue the HSC defect when added to dissected AGMs in ex vivo explant cultures. In addition, the HSC defect was recapitulated in another mouse model for catecholamine deficiency, in which Th was deleted, and addition of a Th inhibitor to wild-type AGM explant cultures had a profound negative effect on HSC numbers.

Previously, an influence of the SNS on HSC behavior in the adult system has been documented. It was shown that catecholamines regulate HSC proliferation and migration by acting either on the niche or on HSCs directly (Katayama et al., 2006; Méndez-Ferrer et al., 2008; Spiegel et al., 2007). Both the β_2_- and the β_3_-adrenergic receptor were shown to be involved in mediating catecholamine signaling in this context. Our results now extend this functional association between the SNS and the definitive hematopoietic system to the time point when they both develop in close proximity to each other in the AGM region. We found robust expression of the β_2_-adrenergic receptor in the AGM and localized it to HSCs, other hematopoietic cells inside the lumen of the aorta, and endothelial cells on the ventral side of the dorsal aorta. Some of these endothelial cells showed a more rounded appearance, suggesting that these may be hemogenic and/or nascent HSCs and hence implying the action of catecholamines directly on emerging HSCs or their direct precursors. Adrenergic receptor expression was also found on some AGM-derived stromal cell lines with supportive capacity (data not shown), raising the possibility that catecholamines may additionally act on cells of the developing HSC niche. However, adding catecholamines to cocultures of AGM HSCs with these stromal cells did not result in increased repopulation activity recovered at the end of the coculture period (data not shown).

It has been reported that catecholamines can affect the proliferation and mobilization of HSCs (Katayama et al., 2006; Spiegel et al., 2007). The fact that we observed an increase in HSCs as reflected in the number of mice being repopulated with higher individual donor chimerisms and the fact that this can occur in explant-cultured AGMs where migration is unlikely to play a role suggest that the effect catecholamines have on emerging HSCs and/or their precursors also involves induction of proliferation or increased production of HSCs from pre-HSCs. Since HSC expansion in AGM explant cultures has been linked to increased maturation from pre-HSCs rather than HSC proliferation (Taoudi et al., 2008), and since we saw a decrease in intra-aortic clusters and hemogenic endothelial cell numbers in the absence of catecholamines, the latter possibility is more likely. It is also unlikely that catecholamines regulate HSC survival because we saw no increase in apoptosis in AGM HSCs exposed to a Th inhibitor in culture, and catecholamines were unable to maintain sorted AGM HSCs in culture in the absence of stromal support (data not shown).

In addition to its expression in the developing kidney and SNS, Gata3+ cells were detected in the ventral mesenchyme just underneath the dorsal aorta. The identity and function of these subendothelial mesenchymal cells is still highly debated. A number of known regulators of HSCs are expressed in this region, including Bmp4 and Runx1 (Durand et al., 2007; North et al., 1999). It may therefore represent the immediate HSC microenvironment in the AGM. Whether Gata3 plays an additional role in AGM hematopoiesis connected with this expression pattern and whether it interacts with other hematopoietic regulators in this region is currently unknown. The subaortic mesenchyme has also been suggested to contain precursors of HSCs (Bertrand et al., 2005; Rybtsov et al., 2011; Zovein et al., 2008). Although our data suggest that Gata3 may not be expressed in AGM-derived HSCs, we cannot rule out that Gata3 is present in some of their precursors, which has been proposed by other reports (Bertrand et al., 2005; Manaia et al., 2000). However, the observation that *Gata3*^*−/−*^ ESCs can contribute to the myeloid and B lineage in adult mice (Ting et al., 1996) argues against an essential cell-autonomous role of Gata3 in the specification of HSCs from their precursors.

We have previously shown that genes coding for regulators of tissues that develop in the vicinity of the dorsal aorta are upregulated at the time of HSC emergence in the AGM (Mascarenhas et al., 2009). This led us to hypothesize that the process of HSC generation occurs in the context of the development of these other tissues. We have now demonstrated through the analysis of Gata3- and Th-deficient embryos that there is indeed a functional link between the developing hematopoietic and sympathetic nervous system. As with the recently reported role of blood flow in HSC generation, this highlights that HSC emergence in the AGM does not occur in isolation and has to be studied in the context of other developmental processes occurring at the same time.

## Experimental Procedures

### Mice and Embryo Generation

Wild-type C57BL/6, *Th* knockout (Figure S2D and Supplemental Experimental Procedures), *Gata3* knockout (Pandolfi et al., 1995), *Gata3* knockout crossed with *Ly-6A GFP* transgenic (de Bruijn et al., 2002), and *Gata3-LacZ* knockin (van Doorninck et al., 1999) mice were used for timed matings with the day of vaginal plug detection being considered as day 0. For the pharmacological rescue experiments, pregnant *Gata3*^*+/−*^ females were given 100 μg/ml L-phenylephrine (Sigma), 100 μg/ml isoproterenol (Sigma), and 2 mg/ml ascorbic acid (Sigma) in their drinking water starting from day 8.5 after vaginal plug detection until the day the embryos were obtained. All mice were housed according to institute regulations and procedures were carried out in compliance with UK Home Office licenses.

### AGM Explant Cultures

E11–11.5 AGMs were cultured on Durapore filters (Millipore) at the air-liquid interface in M5300 long-term culture medium (Stem Cell Technologies) supplemented with 10^−6^ M hydrocortisone (Sigma). Where indicated, recombinant murine IL3 (BD Biosciences) at 100 ng/ml or the Th Inhibitor α-Methyl-DL-tyrosine methyl ester hydrochloride (Sigma) at 5 mM (Wolkersdorfer et al., 1996) was added to the culture medium. For the in vitro rescue experiments, 10 μM L-phenylephrine (Sigma), 10 μM isoproterenol (Sigma), and 200 μM ascorbic acid (Sigma) was added to the culture medium and the medium was refreshed every day. After 2–4 days, AGMs were dissociated with collagenase (Sigma) and single-cell suspensions were transplanted into irradiated recipients or analyzed by flow cytometry.

### Long-Term Transplantations

AGM cell preparations were intravenously injected into recipients that had received a split dose of 9–9.5 Gy of γ irradiation. After 1 and 4 months, donor contribution to the recipients' peripheral blood was determined by semiquantitative PCR or FACS analysis using antibodies specific to the Ly5.1 or Ly5.2 isoforms. Mice were considered to be repopulated if the donor contribution was at least 10% by PCR or 5% by FACS.

### CFU-S Assays

E10.5 AGM cell preparations were injected at 1 embryo equivalent into irradiated (10 Gy) recipients. Eleven days posttransplantation, the spleens of the recipients were removed and fixed in Bouin's solution and the number of macroscopic colonies per spleen was determined.

### Gene Expression Analysis

Tissues and cells were dissociated in Tri Reagent (Sigma), RNA was isolated and treated with RQ1 DNase I (Promega), and cDNA was prepared using Oligo dT primers and Superscript II reverse transcriptase (Invitrogen). For the analysis of different isolated cell populations, an equal number of each cell type was directly sorted into Tri Reagent. Primers for the quantitative RT-PCR analysis can be found in the Supplemental Experimental Procedures, and expression was normalized to β-actin, *Actb*, and TATA box binding protein, *Tbp*.

### X-gal Staining, Immunohistochemistry, and In Situ Hybridization

*Gata3*^*+/lz*^ embryos were stained with X-gal for β-galactosidase as described previously (Ottersbach and Dzierzak, 2005). Cryosections were then prepared and counterstained with Neutral Red.

The CD34 immunostaining of *Ly-6A GFP+* embryo sections for the quantification of GFP+ cells in the endothelial layer was carried out as described previously (Ling et al., 2004). Using the same protocol, embryo sections were also stained with the following antibodies: Adrb2 (Abcam), Gata2 (Santa Cruz), Gata3 (Absea), Nos3 (Abcam), and Th (Millipore). Secondary antibodies used were anti-rabbit-Alexa 555 (Invitrogen), anti-mouse-Alexa 488 (Invitrogen), biotin-conjugated anti-rabbit antibody (Santa Cruz Biotechnology), biotin-conjugated anti-rat antibody (BD Biosciences), Streptavidin-Cy3 (Stratech Scientific), and Streptavidin-Cy5 (Jackson Immunoresearch). Confocal images were taken on a Zeiss LSM510 META Confocal Microscope (Carl Zeiss Ltd., Wellyn, UK).

For in situ hybridization on cryosections, the following riboprobes were used: *Th* (Mascarenhas et al., 2009); *Gata2*: prepared by RT-PCR from E11 AGM cDNA using the following primers; forward, GCACAATGTTAACAGGCCAC; reverse, AAGTGGGTCTCTTGGGATGG; *Hand1*: prepared by RT-PCR from E11 AGM cDNA using the following primers; forward, TTTGGACGTCTGAACCCTTC; reverse, CTGTGCGTCTCCTCTCCTTC; and *Gfi1*: fragment encompassing nucleotides 2395–2862 (accession number NM 010278). In situ hybridization experiments were performed as described previously (Ottersbach and Dzierzak, 2005). Bright-field images were obtained using a Zeiss AxioSkop2 Wide-Field Microscope (Carl Zeiss Ltd., Wellyn, UK).

### Flow Cytometry Analysis and Cell Sorting

For the detection and analysis of Gata3-expressing cells, collagenase-dissociated *Gata3*^*+/lz*^ AGM cells were loaded with the fluorescent β-galactosidase substrate fluorescein di-β-D-galactopyranoside (FDG, Molecular Probes) by hypotonic shock and kept on ice for 1 hr before cell sorting experiments or 30 min before further antibody staining for flow cytometry analysis. Wild-type AGM cells were loaded with FDG alongside for background control. Antibody staining was performed for 25 min on ice using antibodies specific to CD34, ckit, CD31, CD41, CD45 (all BD Biosciences), VE-Cadherin (eBioscience), Adrb2 (Santa Cruz), and anti-rabbit-Alexa 555 (Invitrogen; for the Adrb2 analysis). Dead cells were excluded via 7-aminoactinomycin D staining (7AAD, Invitrogen).

## Figures and Tables

**Table 1:** Effect of Gata3 Deletion on AGM HSC Numbers

	Genotype	Number of Mice Repopulated/Total Transplanted	Mice Reconstituted (%)	Chimerism (%)
Direct transplantation^a^	*Gata3*^*+/+*^	7/12	(58)	11–89
	*Gata3*^*+/−*^	4/23	(17)	26–85
	*Gata3*^*−/−*^	1/7	(14)	10
After explant^b^	*Gata3*^*+/+*^	4/9	(44)	11–100
	*Gata3*^*+/−*^	10/17	(59)	11–75
	*Gata3*^*−/−*^	1/7	(14)	28

The percentage of chimerism was determined by semiquantitative PCR. See also Figure S1.

a AGMs from *Gata3*^*+/+*^, *Gata3*^*+/−*^, or *Gata3*^*−/−*^ E11 embryos were dissociated with collagenase treatment and directly transplanted as one tissue per recipient (1 embryo equivalent [ee]) into irradiated adult recipients.

b AGMs from *Gata3*^*+/+*^, *Gata3*^*+/−*^, or *Gata3*^*−/−*^ E11 embryos were cultured as explants for 3 days before being dissociated and transplanted (as 1 ee) into irradiated adult recipients.

**Table 2:** HSC Activity in Sorted AGM Cell Populations from E11 *Gata3*^*+/lz*^ Embryos

	Cell Population	Cell Dose (Embryo Equivalent)	Number of Mice Repopulated/Total Transplanted	Chimerism (Percent)
Direct transplantation^a^	Gata3-LacZ+	1–5	0/13	0
	Gata3-LacZ−	1–4	1/14^c^	38
After explant with IL3^b^	Gata3-LacZ+	1–2	0/4	0
	Gata3-LacZ−	1–2	4/4	46–100

The percentage of chimerism was determined by semiquantitative PCR.

a AGMs were dissected from *Gata3*^*+/lz*^ E11 embryos, the urogenital ridges were removed, and the cells were dissociated with collagenase treatment, stained with FDG, sorted into LacZ+ and LacZ− populations, and directly transplanted into irradiated adult recipients.

b AGMs were dissected from *Gata3*^*+/lz*^ E11 embryos and cultured as explants in the presence of 100 ng/ml IL3. After 3–4 days, they were dissociated with collagenase treatment, stained with FDG, sorted into LacZ+ and LacZ− populations, and transplanted into irradiated adult recipients.

c We detected the donor marker in a second recipient. However, since the contribution was below 10%, it was counted as negative.

## Supplemental Information

Document S1. Figures S1–S3 and Supplemental Experimental Procedures
