# Supplementary material for: Signaling from the Sympathetic Nervous System Regulates Hematopoietic Stem Cell Emergence during Embryogenesis
Source: Cell Stem Cell. 2012 Oct 5;11(4):554–66. doi: 10.1016/j.stem.2012.07.002 (PMC3510442; doi:10.1016/j.stem.2012.07.002)
Supplement: Document S1. Figures S1–S3 and Supplemental Experimental Procedures [file mmc1.pdf]

Cell Stem Cell, volume *11*
Supplemental Information

Signaling from the Sympathetic Nervous System Regulates Hematopoietic Stem Cell Emergence during Embryogenesis

Simon R. Fitch, Gillian M. Kimber, Nicola K. Wilson, Aimée Parker, Bahar Mirshekar-Syahkal, Berthold Göttgens, Alexander Medvinsky, Elaine Dzierzak, and Katrin Ottersbach

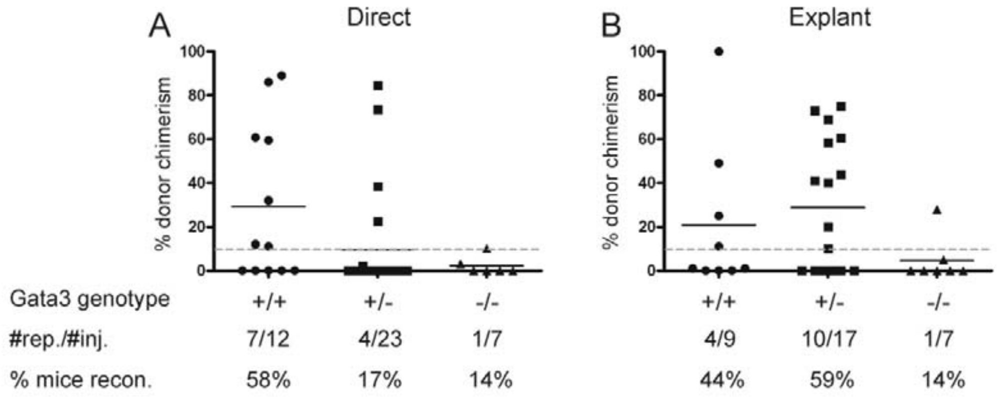

C

Genotype	Number of Positive Recipients/ Total Recipients	CFU-S_{11}/ AGM
Gata3+/+	7/7	7.0
Gata3+/-	15/15	6.4
Gata3-/-	3/8	2.6

Figure S1, related to Table 1: Reconstitution levels and progenitor numbers from *Gata3*$^{+/+}$, *Gata3*$^{+/-}$ and *Gata3*$^{-/-}$ AGM cells.

Scatter plots of the reconstitution levels of individual mice that had received freshly dissected E11.5 AGM cells (**A**) or cells from explant-cultured E11.5 AGMs (**B**) of the given genotypes. Solid lines represent the mean percent chimerism. Dashed lines indicate the threshold above which mice are being considered positive for repopulation (10%). The number of positive mice per total number of injected mice (#rep./#inj.) is stated underneath. This ratio is also expressed as a percentage (% mice recon.). (**C**) AGMs from *Gata3*$^{+/+}$, *Gata3*$^{+/-}$ or *Gata3*$^{-/-}$ E10.5 embryos were dissociated with collagenase treatment and directly transplanted (as 1ee) into irradiated adult recipients. CFU-S_{11} colonies were scored in positive recipients and are expressed as the number of CFU-S_{11} colonies per AGM injected.

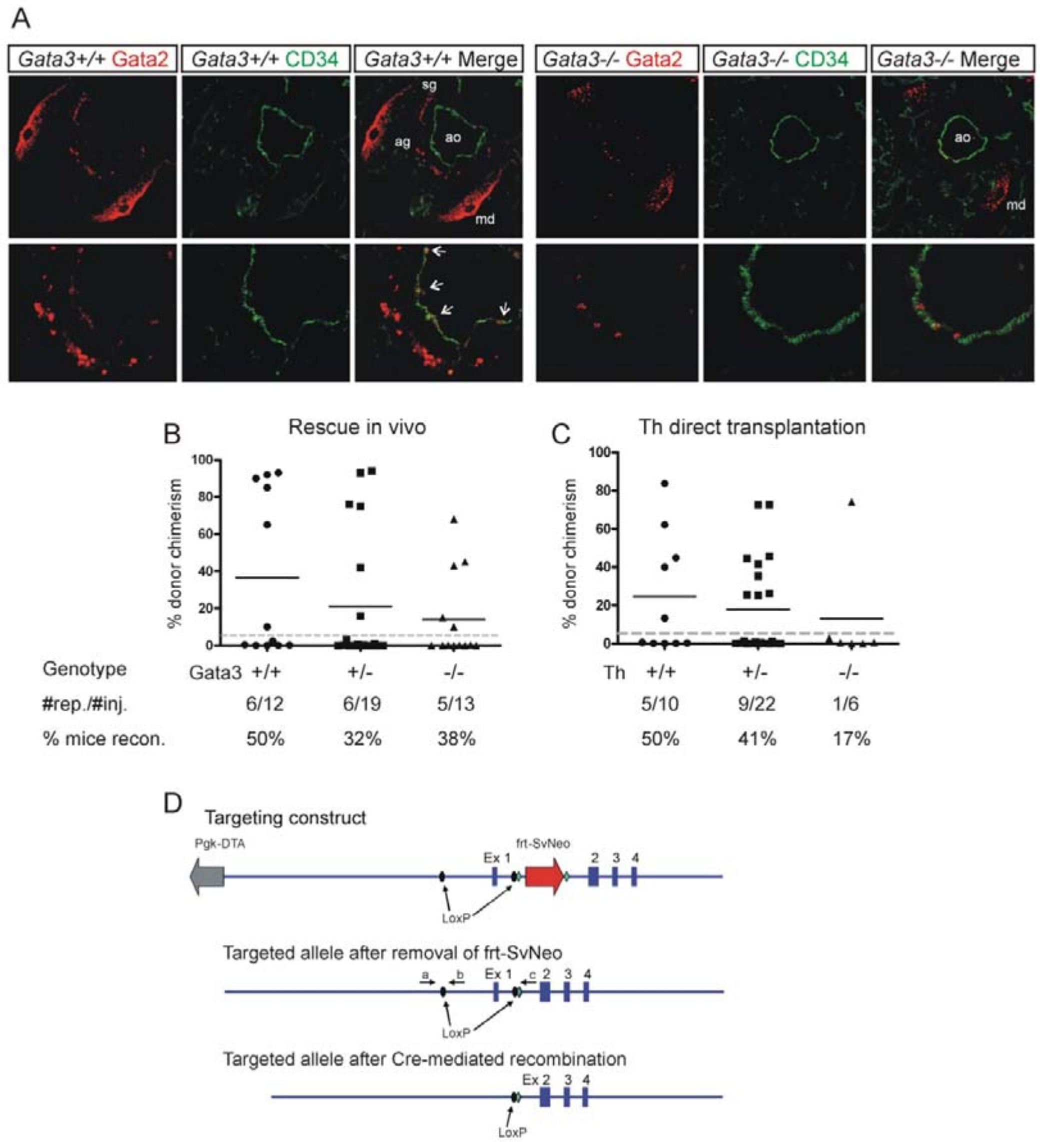

Figure S2, related to Figure 4A, B & C: Disruption of the sympathetic nervous system results in defective HSC production in the AGM.

(**A**) Immunostaining for Gata2 (red/Alexa555) and CD34 (green/FITC) on sections from E11.5 *Gata3*$^{+/+}$ and *Gata3*$^{-/-}$ embryos with close-up views of the aorta shown underneath. Ventral, down. Arrows highlight intra-aortic clusters. Images were taken using a Zeiss LSM510 META Confocal Microscope (Carl Zeiss Ltd., Wellyn, UK). ag, adrenal anlage; ao, dorsal aorta; md, mesonephric duct; sg, sympathetic ganglia. (**B**) Scatter plot of the reconstitution levels of individual mice that had

received freshly dissected *Gata3*$^{+/+}$, *Gata3*$^{+/-}$ and *Gata3*$^{-/-}$ AGM cells from embryos that had been treated with catecholamine derivatives in the drinking water from E8.5 or (**C**) that had received freshly dissected *Th*$^{+/+}$, *Th*$^{+/-}$ and *Th*$^{-/-}$ AGM cells. The genotype of the injected AGM cells is given underneath. Solid lines represent the mean percent chimerism. Dashed line indicates the threshold above which mice are being considered positive for repopulation (5%). The number of positive mice per total number of injected mice (#rep./#inj.) is stated underneath. This ratio is also expressed as a percentage (% mice recon.). (**D**) Outline of the targeting strategy used for the generation of the Tyrosine Hydroxylase knockout mice.

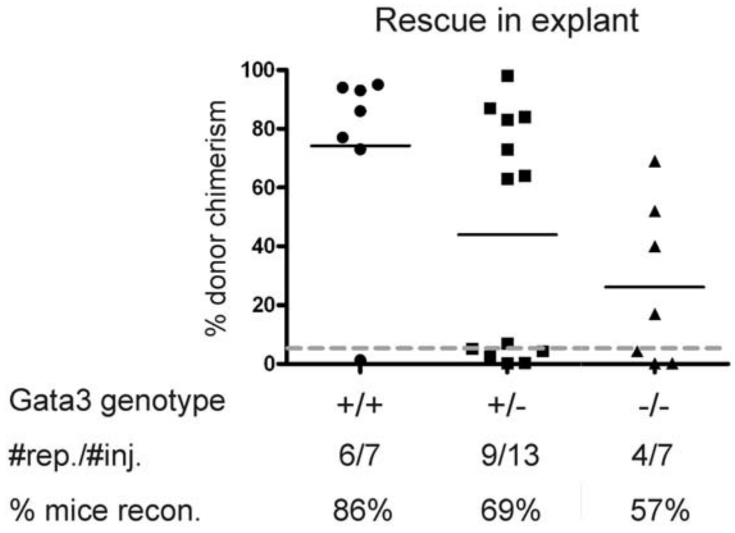

Figure S3, related to Figure 5D: Reconstitution levels with *Gata3*$^{+/+}$, *Gata3*$^{+/-}$ and *Gata3*$^{-/-}$ AGM cells cultured in the presence of catecholamine derivatives.

Scatter plot of the reconstitution levels of individual mice that had received cells from E11 AGMs that had been cultured in the presence of catecholamine derivatives. The genotype of the injected AGM cells is given underneath. Solid lines represent the mean percent chimerism. Dashed line indicates the threshold above which mice are being considered positive for repopulation (5%). The number of positive mice per total number of injected mice (#rep./#inj.) is stated underneath. This ratio is also expressed as a percentage (% mice recon.).

SUPPLEMENTAL EXPERIMENTAL PROCEDURES

Generation of Tyrosine Hydroxylase knock-out mice

Tyrosine Hydroxylase-deficient mice (a kind gift from Dr Richard Palmiter) were generated by inserting a loxP site into the *Bsa*B1 site located ~ 600 bp 5' of the transcription start site of the *Th* gene (**Fig. S2D**). Another loxP site and a frt-flanked *SvNeo* selectable gene were inserted into the Kpn1 site in the first intron of the *Th* gene. The targeting construct had ~ 12 kb of 5' flanking sequence and 7 kb of 3' flanking sequence as well as a *Pgk-DT*$_A$ gene for negative selection. Linearized DNA was electroporated into AB1 embryonic stem cells. Correctly targeted events (~20% of total) were detected by Southern blot of *EcoR*1-digested DNA using a probe just 3' of the short arm of the targeting construct. One clone transmitted the targeted allele through the germline. Mice bearing the targeted allele were bred with FLPer mice expressing the FLIP recombinase (Farley et al., 2000) to remove the *frt-Sv-Neo* gene. After removing the FLPer gene, the mice were backcrossed to C57Bl/6 mice for many generations. Exon 1 and the promoter were deleted using the Mox2-Cre mouse line and heterozygous mice backcrossed to C57BL/6 to remove the Mox2-Cre from the background. *Th*$^{+/-}$ mice were bred to obtain E11.5 embryos of all three genotypes, which were determined by using primers a, b and c (**Fig. S2D**).

Quantitative RT-PCR primer sequences

Actb	forward:	TCCTGGCCTCACTGTCCA
	reverse:	GTCCGCCTAGAAGCACTTGC
Adra1d	forward:	GCCATCGTCGTGGGTGTCTTCG
	reverse:	GCGCGCTTGAACTCGCGAC

Adrb2	forward:	AGATTCCACGCCCAAA
	reverse:	TGGAGGACCTTCGGAGT
Adrb3	forward:	TCCGTCGTCTTCTGTGTAGC
	reverse:	CCTTCATAGCCATCAAACCTG
C-myb	forward:	CGAAGACCCTGAGAAGGAAA
	reverse:	GCTGCAAGTGTGGTTCTGTG
Bmp4	forward:	AGCGTCCCGCCAGCCGA
	reverse:	CGGAGCTCTGCCGAGGAG
Gata2	forward:	TGGCAGCAGTCTCTTCCAT
	reverse:	ACACACTCCCGGCCTTCT
Gata3	forward:	TACGGAAACTCCGTCAGGGC
	reverse:	AAGGGGCTGAGGTTCCAGGG
Gfi1	forward:	TCCGAGTTCGAGGACTTTTG
	reverse:	CATGCATAGGGCTTGAAAGG
Nos3	forward:	CAGGACAACCTCATCCCTGT
	reverse:	GCCTTCTGCTCATTTTCCAG
Runx1	forward:	CGGAGGGAAACTGTGAATGC
	reverse:	CCCAAAGCTGTAGCTGTCTC
Tbp	forward:	GAATATAATCCCAAGCGGTTTG
	reverse:	ACTTCACACACAGCTCCCC
VE-Cadherin	forward:	CAAGATGCCCTGGGTCTG,
	reverse:	CACCGAAAATGTGTATGTGGA

REFERENCES

Farley, F.W., Soriano, P., Steffen, L.S., and Dymecki, S.M. (2000). Widespread recombinase expression using FLPeR (flipper) mice. Genesis *28*, 106-110.
